# Plummer-Vinson syndrome

**DOI:** 10.1186/1750-1172-1-36

**Published:** 2006-09-15

**Authors:** Gottfried Novacek

**Affiliations:** 1 Medical University of Vienna, Department of Internal Medicine IV, Division of Gastroenterology and Hepatology, Waehringer Guertel 18-20, A-1090 Vienna, Austria

## Abstract

Plummer-Vinson or Paterson-Kelly syndrome presents as a classical triad of dysphagia, iron-deficiency anemia and esophageal webs. Exact data about epidemiology of the syndrome are not available; the syndrome is extremely rare. Most of the patients are white middle-aged women, in the fourth to seventh decade of life but the syndrome has also been described in children and adolescents. The dysphagia is usually painless and intermittent or progressive over years, limited to solids and sometimes associated with weight loss. Symptoms resulting from anemia (weakness, pallor, fatigue, tachycardia) may dominate the clinical picture. Additional features are glossitis, angular cheilitis and koilonychia. Enlargement of the spleen and thyroid may also be observed. One of the most important clinical aspects of Plummer-Vinson syndrome is the association with upper alimentary tract cancers. Etiopathogenesis of Plummer-Vinson syndrome is unknown. The most important possible etiological factor is iron deficiency. Other possible factors include malnutrition, genetic predisposition or autoimmune processes. Plummer-Vinson syndrome can be treated effectively with iron supplementation and mechanical dilation. In case of significant obstruction of the esophageal lumen by esophageal web and persistent dysphagia despite iron supplementation, rupture and dilation of the web are necessary. Since Plummer-Vinson syndrome is associated with an increased risk of squamous cell carcinoma of the pharynx and the esophagus, the patients should be followed closely.

## Disease name and synonyms

Plummer-Vinson syndrome, PVS

Paterson-Kelly syndrome

Paterson-Brown Kelly syndrome

Sideropenic dysphagia

## Definition

Plummer-Vinson syndrome is defined by the classic triad of dysphagia, iron-deficiency anemia and esophageal webs. Even though the syndrome is very rare nowadays, its recognition is important because it identifies a group of patients at increased risk of squamous cell carcinoma of the pharynx and the esophagus.

## History

The syndrome eponym has been frequently discussed [1,2]. The most used name is Plummer-Vinson syndrome, named after Henry Stanley Plummer (1874–1936) and Porter Paisley Vinson (1890–1959) who were physicians on the staff of the Mayo Clinic. In 1912, Plummer reported a series of patients with long-standing iron deficiency anemia, dysphagia and spasm of the upper esophagus without anatomic stenosis, which was described as hysterical [3]. In 1919 Vinson reported another case of 'angulation' of the esophagus and attributed the first description of this entity to the earlier report of Plummer [4]. Some years later he published a series of patients with dysphagia who were successfully treated with the passage of bougies; most of the patients were women [5].

Another term is Paterson-Kelly syndrome, named after Donald Ross Paterson (1863–1939) and Adam Brown-Kelly (1865–1941), both British laryngologists, who published their findings independently in 1919 [6,7]. They were the first to describe the characteristic clinical features of the syndrome. Paterson gave the fullest description but without reference to anemia. He was also the first to draw attention to an association with post-cricoid carcinoma. Brown-Kelly not only described the signs and symptoms of the condition, but also considered anemia.

## Epidemiology

Exact data about incidence and prevalence of the syndrome are not available. In the first half of the 20th century Plummer-Vinson syndrome seemed to be common in Caucasians of Northern countries, particularly among middle-aged women [8]. Nowadays, it is extremely rare. In a series of 1000 consecutive patients who underwent a cineradiographic examination of the hypopharynx and cervical esophagus, webs were found in 5.5% of the cases but only six patients had dysphagia attributable to the webs, and none of the patients fulfilled the criteria for Plummer-Vinson syndrome [9]. Only case reports (rather than series of patients) have been published in the literature in recent years. The rapid fall in the prevalence of the syndrome correlates with the improvement of nutritional status and disappearance of the widespread iron deficiency in countries where the syndrome had been previously described [10]. In Africa, where both iron deficiency and malnutrition are common, the syndrome is very rare.

## Clinical description

The main clinical features of Plummer-Vinson syndrome are postcricoid dysphagia, upper esophageal webs and iron deficiency anemia. Most of the patients are white middle-aged women, in the fourth to seventh decade of life [8,11] but the syndrome has also been described in children and adolescents [12-14]. The dysphagia is usually painless and intermittent or progressive over years, limited to solids and sometimes associated with weight loss. Symptoms resulting from anemia such as weakness, pallor, fatigue and tachycardia may dominate the clinical picture. Furthermore, it is characterized by glossitis, angular cheilitis and koilonychia (spoon-shaped finger nails). Enlargement of the spleen and thyroid may also be observed [11].

Plummer-Vinson syndrome has been identified as a risk factor for developing squamous cell carcinoma of the upper gastrointestinal tract. Three to 15 per cent of the patients with Plummer-Vinson syndrome, mostly women between 15 and 50 years of age, have been reported to develop esophageal or pharyngeal cancer [15,16]. A decreasing trend in the overall incidence of hypopharyngeal cancer in women was demonstrated, probably due to diminished prevalence of Plummer-Vinson syndrome [17].

### Recent case reports

Analysis of English language case reports published in the literature during the last 7 years (1999–2005) revealed that 25 out of the 28 adult patients with Plummer-Vinson syndrome were women (89 %) [18-34]. The mean age at presentation was 47 years (range 28–80 years). All patients had an iron deficiency anemia with a mean value of hemoglobin of 8.2 g/dL. Celiac disease was the most frequently reported associated disorder (n = 6) [19,20,22,27,29], followed by increased menstrual blood loss (n = 5), and hiatus hernia and chronic gastrointestinal bleeding of unknown origin (n = 3) [25,27,28,30,31,33]. One patient was a multiparous woman, one patient had rheumatoid arthritis and one was reported to have lymphangiomatous macroglossia [24,26,33]. Two patients received transfusions of packed red blood cells, all other patients were treated with iron supplementation. Most of the patients underwent a dilation of the esophageal web (n = 20), in one case it was disrupted endoscopically using the biopsy forceps [23] and in one case a web resection under microlaryngosurgery was performed [18]. In general, the prognosis was very good. The patients were free of relevant dysphagia during the follow-up period. In two cases, an associated squamous cell cancer was diagnosed [20,29]. And in one case a gastric cancer was found by endoscopy one year after the diagnosis of Plummer-Vinson syndrome [34]. This malignancy seems to be extremely rare in patients with Plummer-Vinson syndrome but has been described in two case reports in previous years [35,36].

## Diagnosis

The diagnosis is based on the evidence of iron-deficiency anemia and one or more esophageal webs in a patient with postcricoid dysphagia. Esophageal webs can be detected by barium swallow X-ray but the best way for demonstration is the videofluoroscopy [11,37]. Webs are also detectable by upper gastrointestinal endoscopy. They appear smooth, thin, and gray with eccentric or central lumen. The webs typically occur in the proximal part of the esophagus and may be missed and accidentally ruptured unless the endoscope is introduced under direct visualization [11].

The esophageal webs, which can also occur in the absence of anemia and Plummer-Vinson syndrome, are characterized by one or more thin horizontal membranes consisting of squamous epithelium and submucosa. They usually protrude from the anterior wall, extending laterally but not to the posterior wall, which means that they rarely encircle the lumen.

## Laboratory tests

Laboratory examinations typically reveal iron deficiency anemia with decreased values of hemoglobin, hematocrit, mean corpuscular volume, serum iron and ferritin, and increased total iron binding capacity. Further laboratory abnormalities are usually not described.

## Differential diagnosis

Since dysphagia is a main clinical feature of Plummer-Vinson syndrome, the differential diagnosis includes all other causes of dysphagia especially malignant tumors, benign strictures or esophageal rings. Other reasons for dysphagia are diverticula, motility disorders such as achalasia, spastic motility disorders, scleroderma, diabetes mellitus, gastroesophageal reflux disease, and neuromuscular and skeletal muscle disorders.

## Etiology and pathogenesis

The pathogenesis of Plummer-Vinson syndrome is unknown. The most important possible etiological factor is iron deficiency. This theory is primarily based on the finding that iron deficiency is a part of the classic triad of Plummer-Vinson syndrome together with dysphagia and esophageal webs and that dysphagia can be improved by iron supplementation. Indeed, impaired esophageal motility has been described in Plummer-Vinson syndrome and it was corrected by iron treatment [38,39]. It has been shown that iron deficiency can precede dysphagia [39]. On the other hand, the alimentary tract is susceptible to iron deficiency; it rapidly loses iron-dependent enzymes due to its high cell turnover, which is speculated to cause mucosa degeneration and web formation. However, large clinical series suggested that for many patients iron deficiency is neither a necessary nor a sufficient cause of web formation [9,15].

Other etiologic factors including malnutrition, genetic predisposition or even autoimmune processes have been proposed. The latter is based on the association between Plummer-Vinson syndrome and certain autoimmune disorders such as celiac disease (which was the most frequently mentioned associated disease in the case reports published in recent years), thyroid disease and rheumatoid arthritis [19,20,22,26,27,29,40].

## Management and treatment

The first step in the management of Plummer-Vinson syndrome is to clarify the cause of iron deficiency in order to exclude active hemorrhage, malignancy or celiac disease.

Plummer-Vinson syndrome can be treated easily and effectively with iron supplementation and mechanical dilation. Iron supplementation alone can resolve dysphagia in many patients [11]. However, in case of significant obstruction of the esophageal lumen by esophageal web and persistent dysphagia despite iron supplementation, rupture and dilation of the web should be performed. After endoscopic placement of a guidewire, dilators with a diameter of up to 17 mm can be used [41]. Usually only one dilation is enough to relieve dysphagia, but occasionally multiple sessions are required. Also successful balloon dilation has been described [33]. Since the Plummer-Vinson syndrome is associated with an increased risk of squamous cell carcinoma of the pharynx and the esophagus, the patients should be followed closely. A surveillance upper gastrointestinal endoscopy is recommended every year, even though the effectiveness of this recommendation is not definitively confirmed [11].

## Prognosis

Prognosis of the Plummer-Vinson syndrome is excellent. As described above, dysphagia and anemia can be treated effectively. In case of an associated squamous cell carcinoma of the hypopharynx or upper esophagus the prognosis worsens dramatically.
